# Adversarial Noise Isolation in Multimodal Perception: A Computational Framework Inspired by Inhibitory Control

**DOI:** 10.3390/brainsci16060591

**Published:** 2026-05-30

**Authors:** Weichen Dai, Xingyu Li, Zeyu Wang, Pengbo Hu, Ningping Li, Ruibao Zhang, Yi Zhou

**Affiliations:** School of Information Science And Technology, University of Science and Technology of China, Hefei 230026, China

**Keywords:** inhibitory control, computational modeling, adversarial training, multimodal perception, emotion perception

## Abstract

**Highlights:**

**What are the main findings?**

- The Multi-modal Information Disentanglement (MInD) framework separates affective cues from task-irrelevant fluctuations using an adversarial noise isolation mechanism conceptually inspired by cognitive inhibitory control.
- This “purification-before-fusion” strategy is empirically associated with competitive performance across standard emotion recognition benchmarks while utilizing simple linear layers for final multimodal integration.

**What are the implications of the main findings?**

- The empirical observations indicate that algorithmic noise suppression facilitates robust multimodal perception, providing an alternative computational methodology to models that primarily focus on complex fusion architectures.
- By observing that filtering latent representations is associated with reduced computational complexity required for subsequent integration, the research suggests that algorithmic noise isolation serves as a viable strategy for improving the efficiency of multimodal processing systems.

**Abstract:**

**Background:** Robust perception involves processing heterogeneous sensory signals, such as facial expressions, vocal prosody, and language, particularly in noisy environments. In computational modeling, a key challenge is integrating these diverse inputs while actively filtering uninformative variations. While recent deep learning models address this integration through complex fusion architectures, they typically aggregate features without explicit filtering modules analogous to inhibitory control. In this study, we propose Multi-modal Information Disentanglement (**MInD**), a computational framework designed to test the hypothesis that algorithmic noise isolation facilitates robust multisensory integration. **Methods:** Drawing conceptual inspiration from cognitive theories of modularity, our model decomposes sensory inputs into amodal (modality-invariant) and modal-specific pathways. Furthermore, we introduce an adversarial noise isolation mechanism to serve as an algorithmic analog to cognitive inhibition. Given that our model operates on pre-extracted high-level features, this mechanism functions to isolate latent distributional variance—uninformative fluctuations that persist after initial feature extraction—guiding the network to separate task-relevant affective cues from irrelevant feature variance. **Results:** Empirical evaluations on standard emotion recognition benchmarks indicate that this purification-before-fusion strategy is associated with competitive performance and stability across multiple metrics. Notably, the framework attains these results using simple linear integration layers, suggesting that separating representations prior to fusion may reduce the computational complexity required for subsequent integration. **Conclusions:** These observations highlight the computational utility of algorithmic noise suppression, illustrating how cognitive inspiration can inform efficient machine learning architectures without claiming direct neurobiological validation.

## 1. Introduction

Emotion perception in naturalistic contexts involves multiple modalities. For example, during face-to-face communication, heterogeneous sensory signals such as linguistic semantics, acoustic prosody, and facial expressions are integrated to infer a speaker’s intent. A recurring challenge in both computational modeling and machine learning is addressing this integration process given the inherent heterogeneity of sensory channels and uninformative variance [1]. While multimodal learning systems can leverage complementary signals to improve performance [2], they face the computational challenge of separating task-relevant affective cues from irrelevant sensory fluctuations.

**Figure 1:**
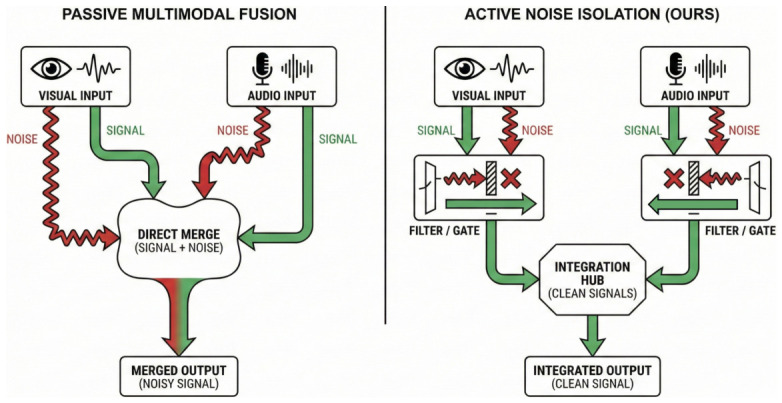
Conceptual Comparison: Passive Accumulation vs. Active Inhibition. (**Left**) Standard fusion approaches typically aggregate inputs without explicit separation, a process where uninformative variance (Red) may propagate into the integrated representation. (**Right**) The MInD framework introduces an active noise isolation module. Drawing conceptual inspiration from Broadbent’s filter model and the information processing principles of inhibitory control, this algorithmic gate functions to attenuate modality-specific variance while routing isolated affective signals (Green) to the integration hub.

Computational models in Multi-modal Sentiment Analysis (MSA) frequently focus on designing fusion architectures to integrate features from different modalities [3]. However, these approaches primarily aggregate information (Figure 1). Drawing conceptual inspiration from cognitive mechanisms like selective attention and inhibitory control, which filter irrelevant stimuli [4,5], it is notable that many existing MSA models process entangled features without explicit mechanisms to suppress uninformative signals. Processing unpurified inputs can result in redundant representations, potentially limiting the robustness of affective perception in noisy contexts.

To improve computational robustness, prior works have managed information flow through representation disentanglement [6,7]. Conceptually inspired by cognitive theories of modularity [8], these approaches separate processing into modality-invariant and modality-specific pathways. However, current disentanglement methods in MSA often rely on factorization constraints or translation mechanisms [9] that may not fully isolate the separated components. Furthermore, they generally do not incorporate active noise isolation. Inspired by the information processing principles that efficient processing involves filtering out irrelevant data, our approach addresses the propagation of uninformative variance into the final integration stage.

In this study, we propose the Multi-modal Information Disentanglement (MInD) framework, a computational architecture designed to investigate algorithmic noise suppression in affective computing. MInD addresses sensory heterogeneity through two conceptually inspired computational processes: (1) Disentanglement, which decomposes inputs into modality-invariant (shared semantics) and modality-specific (stylistic cues) components; and (2) Adversarial Noise Isolation, an algorithmic mechanism conceptually inspired by cognitive inhibition. By optimizing the model to distinguish between task-relevant signals and a generated Gaussian noise prior which serves as a computationally tractable heuristic for latent variance, we implement a filtering step that separates latent representations prior to integration.

Our contributions are summarized as follows:

- We propose an information-theoretic disentanglement framework conceptually inspired by the distinction between amodal and modal-specific information, addressing feature heterogeneity in multimodal computational models.
- We introduce an Adversarial Noise Isolation mechanism. This approach provides an algorithmic analog for the suppression of uninformative variance, evaluating its association with robust emotion perception in MSA.
- Through experiments on standard benchmarks, our empirical observations indicate that this purification-before-fusion strategy is associated with competitive performance using simple linear integration layers, illustrating the potential computational utility of noise filtering prior to multimodal fusion.

## 2. Related Works

### 2.1. Computational Models of Multisensory Binding

In cognitive science, the binding problem concerns how biological systems integrate heterogeneous sensory streams, e.g., visual faces and auditory voices, into a unified perceptual representation. In the computational domain of MSA, early attempts to approximate this integration focused on complex fusion mechanisms. For instance, Tensor Fusion Networks [3] model the interactions between modalities via outer products, serving as an algorithmic analog to holistic integration. Later approaches employed graph neural networks to capture inter-modal dynamics [9]. More recently, attention-based architectures [10], such as the Multimodal Transformer [11], have become prevalent. These models leverage cross-modal attention to dynamically weight inputs, drawing conceptual inspiration from exogenous attention mechanisms in perception. However, a structural divergence remains between these computational models and their cognitive inspirations: while biological information processing principles employ selective attention to filter task-irrelevant variance [12], existing fusion-centric methods typically aggregate entangled features. They often process all inputs indiscriminately, which may lead to representations containing uninformative variations, potentially limiting computational robustness.

### 2.2. Modularity and Inhibitory Control in Representation Learning

Cognitive theories of modularity [8] suggest that information processing involves both specialized (domain-specific) and central (domain-general) systems. In computational modeling, this paradigm provides conceptual inspiration for disentanglement learning, particularly through shared–private frameworks. Models like MISA [6] and FDMER [7] separate representations into modality-invariant and modality-specific components. While this approach addresses modality heterogeneity by isolating unique affective cues from shared semantics, it generally does not incorporate filtering mechanisms analogous to Inhibitory Control [5]. Conceptually inspired by information processing principles, which involve not only the enhancement of relevant signals but also the suppression of irrelevant stimuli, our work introduces an algorithmic analog to this selection mechanism. By implementing an adversarial noise isolation module, we provide a computational heuristic for the suppression of uninformative variance, facilitating the separation of affective information within the latent representation space.

### 2.3. Robustness and Uncertainty in Multimodal Learning

Achieving computational robustness against uninformative variance is a pervasive challenge across diverse multimodal domains. Recent advancements have explored various strategies to mitigate the impact of corrupted modalities. For instance, in autonomous perception, adaptive cross-modal frameworks have been proposed to refine inputs using semantic guidance from cleaner modalities [13]. Similarly, in multimodal recommendation, uncertainty-aware fusion mechanisms dynamically adjust modality weights based on quantified input variance, applying penalties to less reliable features during integration [14]. Furthermore, active inference frameworks have been adapted for tasks like few-shot action recognition to dynamically select modalities based on evidence-based preferences [15].

While these methods are empirically associated with enhanced robustness, they adopt distinct methodologies compared to the framework of MInD. Existing approaches primarily utilize re-weighting or modality dropping. They frequently model uninformative variance as a scalar penalty or discard the modality entirely at the integration stage, which may inadvertently reduce task-relevant, modal-specific cues. In contrast, MInD utilizes an alternative structural approach. Rather than applying penalties or dropping modalities during late-stage fusion, MInD incorporates a generative adversarial noise isolation mechanism prior to integration. By employing a Gaussian prior and mapping the variance into an independent subspace using a Gradient Reversal Layer, MInD filters the latent representations. This methodology is designed to retain the heterogeneous multimodal structure and modal-specific features, serving as an algorithmic analog to active inhibition without discarding sensory channels.

## 3. Methods

### 3.1. Base Architecture

**Figure 2:**
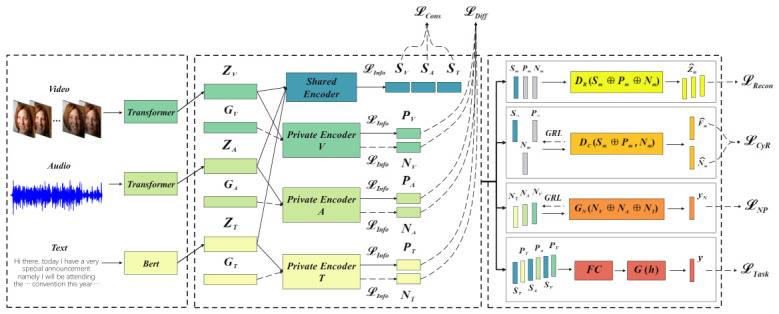
Our Framework. Drawing conceptual inspiration from multisensory integration, the computational model processes information through two functional streams: an Amodal Pathway (implemented via the Shared Encoder) to extract cross-modal semantics ($S_{m}$), and a Modal-Specific Pathway (implemented via Private Encoders) to extract unique sensory cues ($P_{m}$). Conceptually inspired by inhibitory control, we introduce an Adversarial Noise Isolation mechanism to serve as an algorithmic analog: a Gaussian noise prior ($G_{m}$) is fed into the private encoders to approximate uninformative variance ($N_{m}$), facilitating the separation of this variance from task-relevant representations. Finally, the filtered latent representations are integrated ($L_{Task}$) while structural constraints are applied via reconstruction and independence objectives ($L_{Recon},L_{CyR},L_{Diff}$).

Drawing conceptual inspiration from information processing principles, we propose **MInD** as a computational framework to process heterogeneous sensory inputs. We consider a multimodal scenario with visual ($X_{V}$), audio ($X_{A}$), and textual ($X_{T}$) channels. Inspired by early sensory processing stages, we first utilize standard pre-trained feature extractors (BERT for text, Transformers for vision and audio) to encode raw sensory signals into high-level latent representations $Z_{m}\in R^{d_{k}}$ for m∈{V,A,T}. Conceptually inspired by the binding problem and cognitive theories of modularity, our framework (Figure 2) disentangles each input $Z_{m}$ into two functional pathways:

1. The Amodal Pathway ($S_{m}$): A shared encoder $E_{S}$ extracts modality-invariant representations ($S_{m}=E_{S}\left(Z_{m}\right)$), designed to approximate abstract semantic concepts consistent across modalities (e.g., the concept of “anger” itself).
2. The Modal-Specific Pathway ($P_{m}$): Private encoders $E_{P_{m}}$ extract modality-specific characteristics ($P_{m}=E_{P_{m}}\left(Z_{m}\right)$), designed to preserve unique modal qualities (e.g., the specific pitch of a voice or the visual features of an image).

### 3.2. Modeling via Noise Isolation

The implementation of Adversarial Noise Isolation serves as a key computational component in our framework. Cognitive theories suggest that information processing relies on inhibitory control—the active suppression of task-irrelevant stimuli [5]. While some disentanglement methods primarily process entangled representations passively into the specific pathway $P_{m}$, we introduce an explicit noise prior to provide an algorithmic analog to this filtering process.

We sample a stochastic vector $G_{m}∼N\left(0,I\right)$ to serve as a computational proxy for uninformative variance. It is critical to clarify that this Gaussian assumption functions strictly as a computational abstraction, rather than a realistic representation of ecological multimodal noise, which is frequently structured and non-Gaussian. Rather than modeling complex real-world interference, the choice of a Gaussian distribution for $G_{m}$ serves as a computationally tractable heuristic for approximating variance in high-dimensional latent spaces. Because our model operates on compressed neural representations ($Z_{m}$) post-extraction, employing a standard Gaussian distribution acts as a common regularization choice. It provides a simple structural boundary that limits the representational capacity of the noise subspace, empirically aiding the network in separating general uninformative variance from task-relevant modal-specific cues without introducing complex inductive biases.

We then optimize the private encoders to map this variance to a dedicated subspace: $N_{m}=E_{P_{m}}\left(G_{m};\theta_{P_{m}}\right)$. Through adversarial training, the model is guided to distinguish between affective signals and random fluctuations. This process provides an algorithmic analog to active filtering, adjusting the resulting representations $P_{m}$ by mathematically isolating and mitigating latent uninformative variances.

### 3.3. Learning Dynamics and Objectives

Our training objective draws conceptual inspiration from the Efficient Coding Hypothesis [16], which posits that biological information processing principles maximize information transmission while minimizing redundancy.

#### 3.3.1. Maximizing Information Gain

We employ a Jensen–Shannon divergence (JSD) estimator [17] to optimize the information flow ($L_{Info}$). This objective maximizes the Mutual Information (MI) between inputs and their corresponding representations. It is designed to encourage the noise projection $N_{m}$ to approximate the properties of the random input $G_{m}$, facilitating the separation of uninformative variance from the task-relevant embeddings.

(1) $$\begin{array}{cc} L_{Info}= & \sum_{m\in \{V,A,T\}}-{\hat{I}}_{\omega_{S},\theta_{S}}^{\left(JSD\right)}\left(Z_{V}⊕Z_{A}⊕Z_{T};S_{m}\right)\\ + & \sum_{m\in \{V,A,T\}}-{\hat{I}}_{\omega_{P_{m}},\theta_{P_{m}}}^{\left(JSD\right)}\left(Z_{m};P_{m}\right)\\ + & \sum_{m\in \{V,A,T\}}-{\hat{I}}_{\omega_{P_{m}},\theta_{P_{m}}}^{\left(JSD\right)}\left(G_{m};N_{m}\right). \end{array}$$

#### 3.3.2. Redundancy Reduction and Independence

To encourage the separated components to function as modular representations, we apply two statistical constraints:

- Consistency ($L_{Cons}$): We align the amodal representations $S_{m}$ across modalities using the Barlow Twins loss [18]. This minimizes redundancy between modalities regarding the shared semantic content.
  (2) $$L_{Cons}=\sum_{\left(S_{m_{1}},S_{m_{2}}\right)}L_{BT}^{S_{m_{1}},S_{m_{2}}}.$$
- Difference ($L_{Diff}$): We minimize the statistical dependence between task-relevant components ($S_{m},P_{m}$) and the noise component ($N_{m}$) using the Hilbert–Schmidt Independence Criterion (HSIC). This constraint is designed to limit the correlation between the filtered representations and uninformative variance.
  (3) $$L_{Diff}=\sum_{\left(R_{1},R_{2}\right)}HSIC\left(R_{1},R_{2}\right),$$
  $\left(R_{1},R_{2}\right)\in \left\{\left(S_{m},P_{m}\right),\left(S_{m},N_{m}\right),\left(P_{m_{1}},P_{m_{2}}\right),\left(P_{m},N_{m}\right)\right\}$.

#### 3.3.3. Reconstruction and Cyclic Integrity

To mitigate information loss during this filtering process, we employ Direct Reconstruction ($L_{Recon}$) and Cyclic Adversarial Reconstruction ($L_{CyR}$). We optimize the model such that the original latent representation $Z_{m}$ can be approximated from the union of its separated parts. Specifically, we use a decoder $D_{R}$ to map the concatenated subspaces (amodal $S_{m}$, modal-specific $P_{m}$, and noise $N_{m}$) back to the input space, minimizing the mean squared error between the original and reconstructed features:

(4) $${\hat{Z}}_{m}=D_{R}\left(S_{m}⊕P_{m}⊕N_{m}\right).$$

The latter uses a Gradient Reversal Layer (GRL) to mitigate information leakage, discouraging the use of the noise subspace to reconstruct the task-relevant signal:

(5) $$\begin{array}{cc} L_{CyR}= & \sum_{m\in \{V,A,T\}}{∥F_{m}-D_{C}\left(GRL\left(N_{m}\right);\theta_{N_{m}}\right)∥}_{2}^{2}\\ + & \sum_{m\in \{V,A,T\}}{∥N_{m}-D_{C}\left(GRL\left(F_{m}\right);\theta_{F_{m}}\right)∥}_{2}^{2}, \end{array}$$

where $D_{C}\left(\cdot ;\theta_{F_{m}}\right)$, $D_{C}\left(\cdot ;\theta_{N_{m}}\right)$ are the decoders for cyclic reconstructions, $F_{m}=S_{m}⊕P_{m}$.

#### 3.3.4. Integration

Finally, the filtered representations are integrated for the downstream task. Because the disentanglement and noise isolation mechanisms address the heterogeneity gap at the representational level, we utilize simple linear fusion layers to produce the final prediction:

(6) $$h=FC(S_{V}⊕S_{A}⊕S_{T}⊕P_{V}⊕P_{A}⊕P_{T}).$$

#### 3.3.5. Semantic Silence in the Noise Pathway

Conceptually inspired by inhibitory control, the framework incorporates an additional adversarial constraint to limit task-relevant content within the isolated variance. We train a diagnostic classifier, $G_{N}\left(\cdot \right)$, which attempts to predict the emotion label solely from the concatenated noise vectors ($N_{V}⊕N_{A}⊕N_{T}$). By applying a GRL during training, the encoders are optimized to maximize this classifier’s error. This process is designed to reduce the statistical dependence between the isolated variance and the affective task, mitigating the propagation of task-relevant information into the isolated subspace.

#### 3.3.6. Global Optimization Objective

The final learning objective is conceptually inspired by an information processing trade-off: the model is optimized to maximize task performance ($L_{Task}$) while subject to structural constraints. The total loss is formulated as:

(7) $$\begin{array}{cc} L_{all}= & \underset{\mathrm{Perception}}{\underset{︸}{L_{Task}}}+\underset{\mathrm{Silence}}{\underset{︸}{L_{NP}}}+\alpha L_{Info}+\beta L_{Cons}\\ + & \gamma L_{Diff}+\lambda \left(L_{Recon}+L_{CyR}\right). \end{array}$$

Our global objective integrates multiple constraints to guide representation learning. To manage the optimization complexity, we consolidate the weighting into four primary hyperparameters (α,β,γ,λ), suggesting that algorithmic noise regulation can be facilitated by a limited set of computational constraints.

## 4. Results

We designed a series of experiments to investigate whether algorithmic noise isolation facilitates robust multimodal emotion perception. Specifically, we examine whether the MInD framework is empirically associated with improved performance and representational clarity compared to baseline models that do not employ explicit noise filtering mechanisms.

### 4.1. Experimental Setup

#### 4.1.1. Datasets

We evaluate our model on three standard benchmarks representing diverse affective tasks: CMU-MOSI [19] and CMU-MOSEI [20] for sentiment analysis, and UR-FUNNY [21] for humor detection. These datasets contain environmental noise and modality heterogeneity, providing a suitable empirical setting to evaluate our approach. We follow the standard protocols for data splits (as shown in MISA [6] and FDMER [7]) and metrics (Acc-7, Acc-2, F1, MAE, Corr).

#### 4.1.2. Implementation

Consistent with computational approaches that prioritize conceptually inspired architectural design over raw feature engineering, we utilize the pretrained model BERT-base-uncased to obtain a 768-dimension embedding for textual features. Specifically, since the original transcripts are not available for our considered UR-FUNNY version, we follow the same procedure as [6] to retrieve the raw texts from Glove [22]. The acoustic features are extracted from COVAREP [23], where the dimensions are 74 for MOSI/MOSEI and 81 for UR-FUNNY. Moreover, we use Facet (https://imotions.com/platform (accessed on 21 December 2023)) to extract facial expression features for both MOSI and MOSEI, and OpenFace [24] for UR-FUNNY. The final visual feature dimensions are 47 for MOSI, 35 for MOSEI, and 75 for UR-FUNNY.

Our model is built on the Pytorch 2.0.1 with one single NVIDIA 3090 GPU, using a fixed random seed 3407. The learning rate for MOSI is 3 × 10^−5^, and the learning rates for MOSEI and UR-FUNNY are both 2 × 10^−5^.

### 4.2. Evaluation

#### 4.2.1. Comparison with Baseline Architectures

To evaluate MInD, we benchmark against a comprehensive suite of baselines representing diverse computational strategies for multisensory integration. We categorize these baselines into three distinct paradigms:

1. Holistic Fusion Models: representing early integration strategies that process modalities as a unified tensor without explicit structural separation. This includes Tensor Fusion Networks (**TFN**) [3] and Low-rank Multimodal Fusion (**LMF**) [25].
2. Modular Disentanglement Models: representing computational approaches inspired by functional specialization. We compare against **MISA** [6], **FDMER** [7], and the recently proposed **DLF** [26], which employ shared–private factorization to isolate modality-specific cues but still lack an algorithmic analog to the inhibitory control mechanism proposed in our work.
3. Attentional and Contrastive Systems: representing recent state-of-the-art approaches that rely on complex feature alignment or contrastive learning strategies. This category includes attention-based models like **BBFN** [27], **AOBERT** [28], **AcFormer** [29], and **TCHFN** [30], as well as contrastive learning frameworks such as **ConFEDE** [31], **Self-HCL** [32], and the recent **MECAM** [33].

Comparing MInD against this wide spectrum allows us to isolate the specific contribution of adversarial noise isolation versus simply increasing model complexity. Furthermore, it is important to contextualize our results alongside recent high-performing models, such as **MMML** [34]. While such approaches report exceptionally high absolute metrics, they operate under a fundamentally different methodological paradigm. Models like MMML often function as heavy-backbone engineering pipelines, relying on massive, modality-specific pre-trained networks (e.g., RoBERTa and Data2Vec) and heavy fusion modules (e.g., 1.6 M parameters in MMML) to maximize empirical gains. Moreover, to circumvent the uninformative variance present in certain sensory channels, these approaches may actively discard the visual modality altogether, effectively reducing the integration task to bimodal (text and audio) processing.

In contrast, MInD is designed as a computational framework drawing conceptual inspiration from information processing principles. To evaluate the architectural contribution of the proposed noise isolation module without confounding variables, MInD utilizes standardized, lightweight feature extractors. Rather than discarding noisy sensory channels, MInD retains and integrates all three modalities (text, audio, and visual) by incorporating its adversarial noise isolation mechanism to filter uninformative variance. This observation suggests a computationally resilient approach to multisensory integration.

#### 4.2.2. Main Performance

As presented in Table 1, MInD is observed to yield consistent performance across all three datasets, showing improvements over prior disentanglement methods (e.g., MISA, FDMER, DLF) on multiple metrics. A detailed comparison with the recent state-of-the-art disentanglement method DLF provides empirical observations regarding our proposed mechanism. While DLF exhibits a marginal advantage in Acc-7 on the CMU-MOSI dataset, MInD yields a lower regression error (MAE) and higher scores in Acc-2, F1-score, and Correlation compared to DLF. These empirical observations align with our initial hypothesis. Representation factorization or fusion approaches (such as those utilized in DLF and FDMER) may remain susceptible to uninformative sensory outliers. While such models might classify the majority of samples into broad categories (yielding a higher Acc-7), the absence of explicit algorithmic noise filtering is associated with larger prediction deviations when processing uninformative variance, corresponding to a higher absolute error.

In contrast, the adversarial noise isolation mechanism in MInD, drawing conceptual inspiration from inhibitory control to serve as an algorithmic analog, separates this irrelevant variance prior to integration. The empirical results suggest that this filtering step mitigates large prediction deviations, thereby being associated with a reduced MAE. From a computational modeling perspective, this indicates that providing an algorithmic analog for the suppression of uninformative variance, alongside separating sensory channels, facilitates robust multimodal perception. The observation that MInD is associated with these results using simple linear integration layers, rather than more complex Transformer-based fusion baselines, suggests that filtering latent representations prior to fusion serves as a viable computational strategy for multimodal integration.

#### 4.2.3. Statistical Robustness and Variance Analysis

While prior literature in MSA often reports performance based on a single optimal initialization, computational models are inherently sensitive to random seeds. To empirically assess whether the performance differences associated with our adversarial noise isolation mechanism extend beyond random variation, we conducted an extended statistical evaluation.

We executed the complete MInD framework across five independent random initializations using fixed hyperparameters optimized under random seed 3407.

While establishing variance empirically requires multiple runs, training deep architectures incurs substantial computational costs. Following common practices in computational modeling reproducibility [35,36], we reported 5 random initializations to estimate the performance distribution while remaining computationally tractable. Table 2 reports the Mean (μ) and the corresponding Margin of Error at the 95% confidence level (CI), computed using the Student’s *t*-distribution (df=4) across all three benchmarks.

The empirical variance indicates stability across initializations. For instance, on the CMU-MOSI dataset, the mean Acc-7 of MInD (46.00) is observed to exceed the MISA baseline (42.30) by an absolute margin of +3.70. Even when considering the lower bound of the 95% CI, the performance of MInD remains higher than that of baseline models such as FDMER. This statistical analysis suggests that the proposed noise isolation strategy, serving as an algorithmic analog for active filtering, is associated with stable empirical performance when compared to standard multisensory integration paradigms.

### 4.3. Empirical Assessment via Structural Ablation

To empirically assess the contribution of specific computational components, we conducted ablation studies.

Table 3 indicates that removing either the amodal ($S_{m}$) or modal-specific ($P_{m}$) pathways is associated with degraded performance.

### 4.4. Evaluating Mechanisms via Progressive Ablation

To evaluate the contribution of specific computational components and examine their individual and combined effects, we conducted a progressive ablation study. Rather than isolating loss functions one at a time, we evaluate the model by incrementally adding structural and algorithmic modules: from basic structural disentanglement to the complete adversarial noise isolation framework.

As detailed in Table 4, we track the performance evolution across four configurations on the CMU-MOSI dataset:

- **Stage 1: Passive fusion with shared–private framework:** We establish the baseline using MISA, which employs a standard shared–private representation framework without an explicit algorithmic noise filtering mechanism.
- **Stage 2: Structural Disentanglement ($S_{m},P_{m}$) + Basic Integration:** We evaluate an ablated variant of MInD where the noise isolation pathway ($N_{m}$) and the corresponding constraints are removed. While structural disentanglement ($S_{m},P_{m}$) is associated with improvements over the baseline, the performance differences are modest. This observation suggests that representation separation alone may not fully mitigate the effects of uninformative variance.
- **Stage 3: Stage 2 + Adversarial Noise injection ($G_{m}$) + Structural Constraints ($L_{Recon},L_{CyR}$):** We introduce the adversarial noise prior ($G_{m}$) and structural constraints ($L_{Recon},L_{CyR}$). The addition of this adversarial separation step corresponds to measurable performance improvements, indicating the computational utility of the noise isolation module.
- **Stage 4: Stage 3 + Semantic Silence ($L_{NP}$):** Finally, we apply the semantic silence constraint ($L_{NP}$), corresponding to the highest observed performance across the tested configurations. This suggests a complementary effect: the algorithmic noise isolation module is associated with greater empirical gains when $L_{NP}$ is applied. $L_{NP}$ serves as a regularization term designed to explicitly discourage task-relevant cues from being mapped into the noise subspace, thereby limiting information leakage during the filtering process.

These empirical observations indicate that without explicit algorithmic mechanisms to isolate and constrain uninformative variance, the integration process remains susceptible to task-irrelevant fluctuations. The results support the hypothesis that algorithmic noise suppression facilitates robust multisensory integration.

### 4.5. Empirical Assessment of Structural Design Choices

To further assess the specific architectural configurations within MInD, we evaluated three structural variants by altering core mechanisms while keeping the global objective intact.

As shown in Table 5, replacing the Gaussian noise vector with a Uniform distribution U(−1,1) was associated with a decrease in empirical performance. This observation suggests that the Gaussian prior provides a more suitable algorithmic constraint for modeling latent variance than the fixed bounds of a uniform distribution.

We evaluated a variant replacing the GRL with a static orthogonal penalty, which minimizes the cosine similarity between the task-relevant signal and uninformative variance. While Acc-7 remained moderate, the MAE was observed to increase to 0.763. This observation suggests that applying a strict orthogonality constraint is associated with alterations in the continuous manifold of affective representations.

When the shared amodal encoder ($E_{S}$) was removed and the model relied on independent encoders constrained by $L_{Cons}$, the Acc-7 was observed to decrease to 42.6. These empirical results indicate the structural utility of hard weight-sharing as an information routing bottleneck. Because a single shared encoder processes all modalities, it is structurally constrained from capturing modality-specific variance, facilitating the separation of such variance into the private pathway ($P_{m}$). Without this shared bottleneck, independent encoders may incorporate uninformative variance into the semantic representation ($S_{m}$). Consequently, the private encoder ($P_{m}$) captures less of the empirical uninformative variance, which corresponds to the generated noise subspace ($N_{m}=E_{P_{m}}\left(G_{m}\right)$) becoming less correlated with the actual input variance. The adversarial isolation mechanism is subsequently observed to be less effective, as it optimizes against an independent variance space while the uninformative sensory variance propagates through the amodal pathway ($S_{m}$).

### 4.6. Computational Complexity Analysis

To provide an empirical assessment of the computational requirements of the MInD framework, we evaluated its parameter counts and Floating Point Operations (FLOPs) against the MISA baseline under the setting of CMU-MOSI. The analysis was conducted assuming a batch size of 2 and sequence lengths of 50.

A characteristic of the MInD architecture is its asymmetric computational profile between training and inference. As shown in Table 6, while MInD requires a larger parameter footprint during training due to the auxiliary networks, these modules are discarded during the testing phase. Consequently, the active parameters required to execute the inference pathway are reduced. The resulting inference FLOPs represent an approximate 16% computational overhead compared to MISA. In the context of the empirically observed performance differences across evaluated metrics (e.g., an associated +4.3% increase in Acc-7 on CMU-MOSI), this asymmetric design allocates the computational requirements of algorithmic noise isolation to the offline training phase. These empirical observations suggest that such a computational approach facilitates inference efficiency suitable for deployment.

### 4.7. Visualizing Representational Geometry

**Figure 3:**
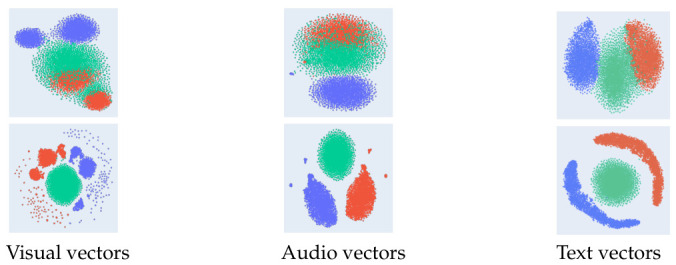
Observation of Representational Clusters. t-SNE visualization of invariant ($S_{m}$, Blue), specific ($P_{m}$, Red), and noise ($N_{m}$, Green) representations on UR-FUNNY. **Top**: Before training (intertwined). **Bottom**: After training. The model is optimized to separate uninformative variance from task-relevant features, which is associated with the formation of distinct representational clusters in the latent space.

We analyze the geometry of the extracted feature space using t-SNE [37]. Figure 3 indicates that MInD separates the latent representation space into observable clusters, where uninformative representations are mapped apart from task-relevant representations. This topological separation suggests that the adversarial training objective functions as a computational heuristic, facilitating the arrangement of the latent space into distinguishable clusters.

To complement the qualitative visual assessment and address the subjectivity of dimensionality reduction, we compute quantitative clustering metrics on the high-dimensional representations of the UR-FUNNY dataset: the Silhouette Score (higher is better, range [−1,1]) and the Davies-Bouldin Index (DBI, lower is better).

As detailed in Table 7, the quantitative metrics align with the visual observations. Prior to adversarial training, the baseline representations exhibit overlap. Following the application of the algorithmic noise isolation mechanism, the Silhouette score is observed to increase to 0.5716, which is consistent with the formation of distinct clusters. Concurrently, the DBI decreases to 1.1473, indicating that the intra-cluster variance of the uninformative representations is constrained, while their spatial distance from the task-relevant affective features is increased. These empirical metrics suggest that the proposed computational framework facilitates the organization of the latent representation space by separating uninformative variance from task-relevant semantic features.

### 4.8. Hyperparameter Sensitivity Analysis

To empirically assess the stability of the proposed MInD framework and examine whether the algorithmic purification-before-fusion strategy is sensitive to specific hyperparameter configurations, we detail our search procedure and present a joint sensitivity analysis.

#### 4.8.1. Search Procedure and Configurations

Due to the computational constraints of processing multimodal sequences, we employed a coarse-to-fine search strategy rather than an exhaustive dense grid search. We initially defined a standard logarithmic search space $\left\{1\times 10^{-4},1\times 10^{-3},1\times 10^{-2},1\times 10^{-1},1\right\}$ for the global objective weights (α,β,γ,λ) evaluated on the validation sets. Subsequently, localized fine-tuning was performed around the initial estimates. The final hyperparameter configurations associated with the lowest validation loss for each dataset are summarized in Table 8.

#### 4.8.2. Joint Sensitivity Analysis

To empirically assess the parameter robustness, we conducted a joint sensitivity analysis on the UR-FUNNY dataset. We systematically varied the core structural constraints: Information Gain weight (α) and Consistency weight (β), across a localized grid adjacent to their optimized values, while keeping other structural constraints fixed.

**Figure 4:**
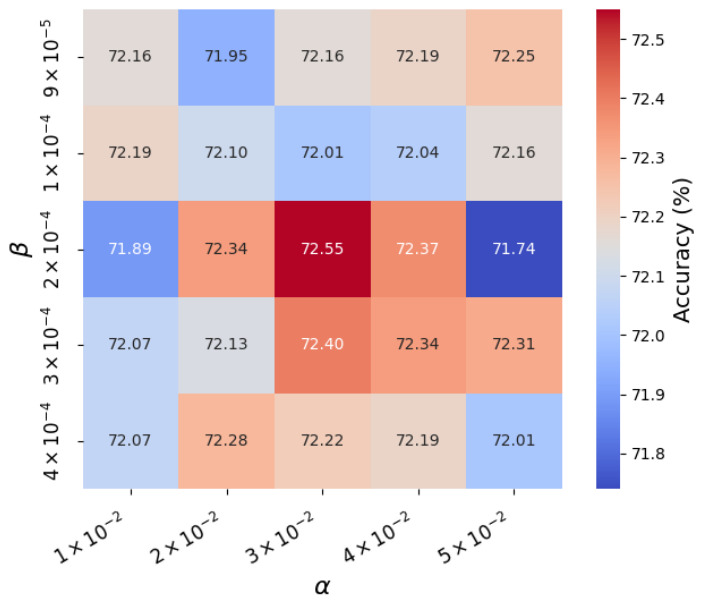
Joint Sensitivity Heatmap on UR-FUNNY. The color gradient represents the Acc-2 performance as α and β vary around their optimums.

As illustrated by the heatmap in Figure 4, the empirical performance landscape is observed to exhibit a region of stability. While the highest observed performance corresponds to $\alpha =3\times 10^{-2},\beta =2\times 10^{-4}$, the framework maintains a consistent Acc-2 across the evaluated grid. In comparison, the baseline shared–private model MISA achieves an Acc-2 of 70.61 on this benchmark. This variance analysis suggests that the empirical performance of the MInD framework is associated with its algorithmic noise isolation architecture, rather than hyperparameter over-tuning.

### 4.9. Cross-Task Generalization to Intent Recognition

An important characteristic of computational models is their applicability across diverse tasks. To empirically assess whether the algorithmic noise isolation mechanism generalizes beyond affective computing, we evaluated MInD on the MIntRec dataset [38] for Intent Recognition.

As shown in Table 9, MInD is observed to yield higher performance metrics compared to baseline methods such as CAGC [39]. This empirical observation suggests that algorithmic noise isolation serves as a versatile computational heuristic applicable to diverse tasks requiring robust multisensory integration, drawing conceptual inspiration from information processing principles.

### 4.10. Preliminary Extension to Scientific Domains

As a preliminary experiment to explore cross-domain generalization, we investigate whether the noise isolation mechanism can be extended to Autoregressive Vision-Language Models (VLMs) in cheminformatics. Standard VLMs frequently utilize a projector to map visual features into the latent representation space of the Large Language Model (LLM), a process that may propagate uninformative visual variance. We evaluate whether the MInD framework can function as a heuristic for noise filtration during this projection stage.

To examine this, we conducted an initial evaluation using a ChemVLM-26B [40] architecture on a subset of the ChemOCR task (mapping molecular images to SMILES strings). The standard projector was replaced with the MInD module. Under restricted computational settings utilizing parameter-efficient tuning, the MInD-Projector processes vision tokens to separate uninformative variance. To guide this separation, the textual representation of ground-truth SMILES strings serves as a semantic anchor to align the visual component.

We generated SMILES strings and computed the average Tanimoto similarity against ground-truth molecules using RDKit. Results averaged over three random initializations are reported in Table 10.

While the absolute scores reflect the restricted nature of this preliminary subset evaluation, the observed relative difference suggests the architectural adaptability of the framework. This initial observation indicates that filtering uninformative variance prior to integration into the LLM represents a potential computational strategy for noise suppression in generative AI for Science applications.

## 5. Discussion

While MInD provides a computational framework to explore the role of algorithmic noise isolation in multimodal perception, several limitations regarding its ecological validity and relationship to biological systems should be noted.

In the current framework, uninformative variance is approximated using a Gaussian prior, which functions strictly as a computationally tractable heuristic and a standard regularization choice. While this serves as an algorithmic proxy for uninformative variance, real-world sensory interference is frequently structured and context-dependent (e.g., background speech in a multi-talker environment, rather than random static). Future iterations of this computational model could incorporate structured variance sources to better approximate the interference observed in naturalistic settings.

MInD operates at Marr’s algorithmic level [41], serving as an algorithmic analog to inhibitory control through adversarial gradients. It does not attempt to approximate the underlying neural implementation, such as inhibitory interneurons or specific cortical feedback loops. While the empirical observations suggest that the model’s performance is associated with functional advantages in algorithmic noise suppression, further research utilizing spiking neural networks or neuroimaging data would be necessary to empirically assess any potential correspondence between this computational abstraction and biological mechanisms.

Multisensory integration in natural contexts is a continuous, dynamic process. Currently, MInD processes segmented sequences. It does not account for the temporal evolution of accumulated variance over prolonged sequences, an aspect that draws conceptual inspiration from information processing principles such as sustained attention. Addressing temporal dynamics remains an area for future computational modeling.

Furthermore, it is necessary to distinguish the structural routing in our framework from generic mathematical regularization. Our structural ablations suggest that adversarial training functions as the algorithmic tool, while the empirically observed performance differences are associated with the structural paradigm conceptually inspired by inhibitory control. When the structural bottleneck (hard weight-sharing) or the semantic silence constraint ($L_{NP}$) is removed, applying generic adversarial regularization is observed to less effectively mitigate the propagation of uninformative variance into the integration process, corresponding to lower empirical performance. This observation indicates that the explicit architectural routing of information, which serves as an algorithmic analog to active isolation, facilitates the empirically observed robustness.

Finally, we acknowledge limitations regarding the reproducibility of such multimodal frameworks. The adversarial optimization facilitated by the GRL involves non-convex dynamics that are observed to exhibit sensitivity to initialization, despite stabilization efforts through hyperparameter configuration across multiple random seeds. Additionally, the framework relies on fixed upstream feature extraction pipelines (e.g., Facet and COVAREP), which contain inherent preprocessing variances. Addressing these reproducibility challenges in future architectures may require transitioning from static feature extractors to approaches that integrate explicit reasoning modules or external domain knowledge directly into the multimodal alignment process. To support algorithmic transparency and reproducibility for the current study, the source code, including data preprocessing pipelines and environment configurations, will be made publicly available at https://github.com/WeeeicheN/MInD (accessed on 17 December 2025) upon publication.

## 6. Conclusions

In this study, we presented the MInD framework as a computational architecture drawing conceptual inspiration from multisensory integration. Our objective was not to provide direct neuroscientific validation, but to investigate whether concepts conceptually inspired by cognitive inhibitory control can be translated into an algorithmic framework for robust multimodal sentiment analysis.

We hypothesized that multisensory integration can be facilitated by an algorithmic filtering heuristic rather than relying solely on additive fusion. To empirically assess this, we implemented an Adversarial Noise Isolation mechanism. By providing an algorithmic analog for the separation between task-relevant signals and uninformative variance through adversarial training, our framework is empirically observed to be associated with competitive performance using simple linear integration layers.

Our empirical observations suggest that the purification-before-fusion paradigm serves as a viable computational strategy. While our framework serves as an algorithmic analog to certain functional outcomes conceptually inspired by top-down filtering, it remains a strict computational abstraction. The framework does not attempt to approximate the underlying neurobiological implementations, such as cortical feedback loops or inhibitory interneuron dynamics.

In summary, this work provides empirical observations supporting the computational utility of algorithmic noise filtering. It suggests that future computational architectures may benefit from exploring latent representation filtering strategies. This approach contributes to the development of resilient multimodal systems that incorporate the algorithmic suppression of uninformative variance, drawing conceptual inspiration from the information processing principles underlying robust multisensory integration.

## Figures and Tables

**Table 1:** Performance comparison on CMU-MOSI, CMU-MOSEI and UR-FUNNY. The proposed MInD framework is empirically observed to exhibit competitive and balanced performance across multiple evaluated metrics. While certain baseline models may achieve higher scores on specific singular metrics (e.g., MECAM on Acc-7), MInD maintains consistent performance across the broader evaluation suite without significant decreases on any individual metric. These empirical observations suggest that the algorithmic noise isolation mechanism facilitates the extraction of generalized affective representations. ‘-’ means the result is not provided in original paper.

Models	CMU-MOSI					CMU-MOSEI					UR-FUNNY
	Acc-7↑	Acc-2↑	F1↑	MAE↓	Corr↑	Acc-7↑	Acc-2↑	F1↑	MAE↓	Corr↑	Acc-2↑
TFN	34.9	80.8	80.7	0.901	0.698	50.2	82.5	82.1	0.593	0.700	68.57
LMF	33.2	82.5	82.4	0.917	0.695	48.0	82.0	82.1	0.623	0.677	67.53
BBFN	45.0	84.3	84.3	0.776	0.755	54.8	86.2	86.1	0.529	0.767	71.68
AOBERT	40.2	85.6	86.4	0.856	0.700	54.5	86.2	85.9	0.515	0.763	70.82
ConFEDE	42.3	85.5	85.5	0.742	0.784	54.9	85.8	85.8	0.522	0.780	-
AcFormer	44.2	85.4	85.2	0.715	0.794	54.7	86.5	85.8	0.531	0.786	-
TCHFN	44.8	86.1	86.3	0.748	0.780	53.2	86.3	86.5	0.538	0.770	-
Self-HCL	-	84.9	85.0	0.711	0.788	-	85.9	85.9	0.531	0.775	-
MECAM	46.6	85.8	85.8	0.715	0.782	52.3	85.2	84.8	0.547	0.748	-
DLF	47.1	85.1	85.0	0.731	0.781	53.9	85.4	85.3	0.536	0.764	-
FDMER	44.1	84.6	84.7	0.724	0.788	54.1	86.1	85.8	0.536	0.773	71.87
MISA	42.3	83.4	83.6	0.783	0.761	52.2	85.5	85.3	0.555	0.756	70.61
**MInD (ours)**	**46.6**	**86.0**	**86.0**	**0.711**	**0.791**	**53.9**	**86.6**	**86.7**	**0.529**	**0.772**	**72.55**

**Table 2:** Statistical Robustness of MInD. Results are reported as Mean ± Margin of Error (95% confidence level) across 5 independent random seeds.

Dataset	Acc-7 ↑	Acc-2 ↑	F1 ↑	MAE ↓	Corr ↑
CMU-MOSI	46.00±1.03	85.68±0.40	85.66±0.42	0.725±0.020	0.787±0.007
CMU-MOSEI	53.42±0.81	86.42±0.30	86.48±0.22	0.531±0.007	0.770±0.002
UR-FUNNY	-	72.29±0.43	-	-	-

**Table 3:** Analysis of Component Necessity. We report MAE and Corr on CMU-MOSI.

Model Variants	CMU-MOSI	
	MAE↓	Corr ↑
**MInD (Full Model)**	**0.711**	**0.791**
Lesioning Disentanglement Modules		
w/o Amodal Pathway ($S_{m}$)	0.793	0.778
w/o Modal-Specific Pathway ($P_{m}$)	0.777	0.773
Lesioning Learning Constraints		
w/o Info. Maximization ($L_{Info}$)	0.755	0.778
w/o Consistency ($L_{Cons}$)	0.789	0.777
w/o Independence ($L_{Diff}$)	0.768	0.769
w/o Reconstruction ($L_{Recon}$)	0.727	0.784
w/o Cyclic Integrity ($L_{CyR}$)	0.787	0.773
w/o Noise Prediction ($L_{NP}$)	0.732	0.783

**Table 4:** Progressive Ablation Study on CMU-MOSI.

Progressive Build-Up	Acc-7 ↑	Acc-2 ↑	F1 ↑	MAE ↓	Corr ↑
Stage 1	42.3	83.4	83.6	0.783	0.761
Stage 2	42.6	84.4	84.5	0.755	0.776
Stage 3	45.3	85.5	85.6	0.732	0.783
Stage 4	**46.6**	**86.0**	**86.0**	**0.711**	**0.791**

**Table 5:** Assessment of Structural Design Choices on CMU-MOSI. Core MInD mechanisms are replaced with standard alternatives.

Structural Variants	Acc-7 ↑	Acc-2 ↑	F1 ↑	MAE ↓	Corr ↑
**MInD (Full Model)**	**46.6**	**86.0**	**86.0**	**0.711**	**0.791**
w/o $G_{m}$ (Replaced with Uniform Noise)	43.8	84.7	84.9	0.748	0.776
w/o GRL (Replaced with Orthogonal Penalty)	45.3	85.0	85.1	0.763	0.773
w/o Shared Encoder (Independent Encoders)	42.6	85.2	85.3	0.761	0.773

**Table 6:** Computational Complexity Comparison. FLOPs are estimated during the inference phase.

Model	Total Params (Training)	Active Params (Inference) *	Inference FLOPs
MISA	∼111.0 M	1.1 M	8.54 G
**MInD (Ours)**	∼151.0 M	2.5 M	9.92 G

* Excludes the heavy, standardized feature extraction backbones (e.g., BERT) common to both.

**Table 7:** Quantitative Separation Metrics on UR-FUNNY. Averaged across three modalities.

State	Silhouette Score ↑	Davies-Bouldin Index ↓
Pre-training (Untrained)	+0.1578	2.9850
Post-training (MInD)	**+0.5716**	**1.1473**

**Table 8:** Final hyper-parameters across datasets. Identified via a coarse-to-fine search strategy based on validation loss.

Dataset	α (Info)	β (Cons)	γ (Diff)	λ (Recon/CyR)
CMU-MOSI	$1\times 10^{-2}$	$1\times 10^{-4}$	$1\times 10^{-1}$	$1\times 10^{-3}$
CMU-MOSEI	$2\times 10^{-2}$	$2\times 10^{-4}$	1	$1\times 10^{-3}$
UR-FUNNY	$3\times 10^{-2}$	$2\times 10^{-4}$	$8\times 10^{-2}$	$8\times 10^{-5}$

**Table 9:** Generalization to MIntRec.

Methods	Acc-20↑	F1 ↑	Pre. ↑	Rec. ↑
MulT	72.52	69.25	70.25	69.24
MISA	72.29	69.32	70.85	69.24
MAG-BERT	72.65	68.64	69.08	69.28
CAGC	73.39	70.09	71.21	70.39
**MInD (Ours)**	**73.71**	**70.12**	**72.34**	**69.66**

**Table 10:** Preliminary Extension to ChemOCR.

Methods	Avg. Sim. ↑
Baseline Projector	33.6
**MInD Projector**	**35.3**

## Data Availability

The data presented in this study are available in CMU-MultiComp-Lab/CMU-MultimodalSDK at https://github.com/CMU-MultiComp-Lab/CMU-MultimodalSDK (accessed on 21 December 2023), reference number [19,20]; ROC-HCI/UR-FUNNY at https://github.com/ROC-HCI/UR-FUNNY/blob/master/UR-FUNNY-V1.md (accessed on 21 December 2023), reference number [21]; thuiar/MIntRec at https://github.com/thuiar/MIntRec (accessed on 21 December 2023), reference number [38]; and lijunxian111/ChemVlm at https://github.com/lijunxian111/ChemVlm (accessed on 2 February 2026), reference number [40].
